# Modeling and experimental verification of a new muffler based on the theory of quarter-wavelength tube and the Helmholtz muffler

**DOI:** 10.1186/s40064-016-3060-1

**Published:** 2016-08-19

**Authors:** Can Wu, Lei Chen, Jing Ni, Jing Xu

**Affiliations:** School of Mechanical Engineering, Hangzhou Dianzi University, Hangzhou, 310018 Zhejiang China

**Keywords:** Low frequency noise, Quarter-wavelength tube, Helmholtz muffler, Noise experiment, SPL

## Abstract

To address the problem of low frequency noise of the internal combustion engine, several existing muffler design methods, such as the theory of the quarter-wavelength tube and the Helmholtz muffler, were examined and compared. This paper proposes a new type of muffler design method, which has the advantages of both the quarter-wavelength tube and the Helmholtz muffler. An example is carried out to illustrate the analysis of original signal, the design of the new muffler and the improvement of the in-car noise. The transmission loss of the new muffler is studied by theoretical method and finite element method. The vehicle test of the new muffler demonstrates excellent performance with a wider noise elimination frequency band and smaller radial size.

## Background

The internal combustion (IC) engine is a major source of automobile noise (Sanjid et al. 2014), and as the standards for automobile noise are becoming increasingly strict, there is greater demand for quieter engines. The process of induction plays a large part in creating engine noise. For some small high-speed machines and some other large turbochargers, the induction noise can reach up to 5 dB (A) higher than the engine noise sometimes (Chiatti et al. 2015). Therefore, it is of great significance to find an approach to control induction noise (Mondal et al. 2014).

Intake noise control begins with the air intake system design, the structure of the valve, cam curve shape, and many other factors, while at the same time, noise control is restrained by power performance and economic efficiency (Boutin and Becot 2015). At present, the actual application of active noise control technology remains in its infancy (Zhou et al. 2009). Therefore, passive noise control is currently the most commonly applied method in the intake noise control, and its core principle is based in muffler design. Mofakhami et al. (2008) studied sound transmission through multilayered viscoelastic air filled cylinders. His result showed that using constant and frequency-dependent viscoelastic material, with high loss factor, leads to uniform noise reduction in the frequency domain. Miandoab et al. (2015) analyzed the nonlinear dynamics and chaotic behavior of nanoresonators with electrostatic forces on both sides, which indicated that the necessary condition for the creation of chaos in the resonator is intersection of the system steady state response with the homoclinic orbit. Ning et al. (2015) used a quasi-one-dimensional model based on the compressible Navier–Stokes equations and a finite volume method to investigate the transient motion of a fluid inside oscillating axisymmetric tubes. Li (2010) developed an objective function to optimize parameters (number and location) of resonator-like cavities, which is based on the tuned weighting coefficient and the acoustic potential energy. Unnikrishnan et al. (2010) presents a simplified modeling approach for numerical simulation of a coupled cavity-resonator system, which was validated by experimentation. It is shown that the resonator volume fraction required to significantly (more than 5 dB) suppress the cavity axial mode. Otherwise, the PZT piezoelectric plates (Li et al. 2013a) and multiple PVDF beam arrays (Li et al. 2013b) are used in the design of the muffler to achieve the purpose of energy collection and recycling.

The resonance-type resistance silencer is based on the principle of the Helmholtz resonator (HR). It has the advantages of simple structure, high amount of noise elimination, and small pressure loss, among other attributes. It is widely used in automotive engine air intake noise control (Sohn and Park 2011). Arefi et al. (2014) was able to improve reverberation time in a conference room using HRs with defined dimensions, diffusers, and sound absorbers. Mao and Pietrzko (2010) carried out an experimental investigation of passive control of sound transmission through a double-glazed window by using an arrangement of HRs. It was shown that a considerable reduction of the transmitted sound pressure levels has been achieved around the mass-air-mass resonance frequency (50–120 Hz). Lee et al. (2013) studied the effect of leakage on the acoustic performance of reactive silencers, such as expansion chambers, HRs, and quarter-wave resonators. Sanada and Tanaka (2013) used two degree of freedom Helmholtz-based resonators with a flexible panel to extend the frequency range of resonant sound absorbers. Yasuda et al. (2013), based on the typical structure, designed a muffler with an interconnecting hole on the tail tube, which was proposed to improve its acoustic performance. Park (2013) introduced a micro-perforated panel of absorbers backed by HRs to improve sound absorption in the low-frequency region, where conventional micro-perforated panel absorbers cannot provide sufficient absorption. Singh and Rienstra (2014) presented a systematic derivation of a solution of the nonlinear HR equation, in order to obtain analytically expressions for impedances close to resonance, while including nonlinear effects. Atak et al. (2014) combined two concepts to design acoustic lenses that are based on HRs. It was shown that using HR-based sonic crystals leads to better acoustic lens designs, especially at the low frequencies, where the local resonances are pronounced. At present, research focuses primarily on designing or analyzing HR, which has a good effects on the air inlet system’s low-frequency noise elimination; however, a larger proportion of intake system noise is high-frequency, especially during rapid acceleration. In order to improve an automobile’s riding comfort, it is necessary to establish a method to suppress the engine’s air inlet high-frequency noise during rapid acceleration.

The air inlet system of traditional engines mainly relies on absorption by the quarter-wavelength straight-tube to suppress high-frequency noise, but two major problems exist. First, the quarter-wavelength straight-tube has a narrow denoising band and cannot cover the low-frequency noise band completely, which results in incomplete noise reduction. Second, due to the greatly restricted radial size of the air inlet tube in the engine’s inlet system, the longer length of the traditional quarter-wavelength straight-tube will be difficult to arrange in a compact car air inlet system. Therefore, in this paper, we propose a new type of muffler design, which has the advantages of both the quarter-wavelength straight-tube and Helmholtz muffler, with a wider noise elimination frequency band and smaller radial size. When used in a practical automobile test, the muffler showed excellent performance in high frequency noise reduction of the air inlet system, and this performance was supported by the experimental data.

## Methods

The principle of the resonance muffler is based on a hole in the tube that connects with the resonance cavity. When the sound wave comes to the resonance structure, the gas will flow back and forth in the hole like a piston reciprocating motion under the influence of the acoustic pressure. Part of the acoustic energy can be consumed into heat energy by the aperture wall friction and damping effects. At present, the main types of resonant silencer are the quarter-wavelength tube and the Helmholtz muffler.

### Quarter-wavelength tube model

**Fig. 1:**
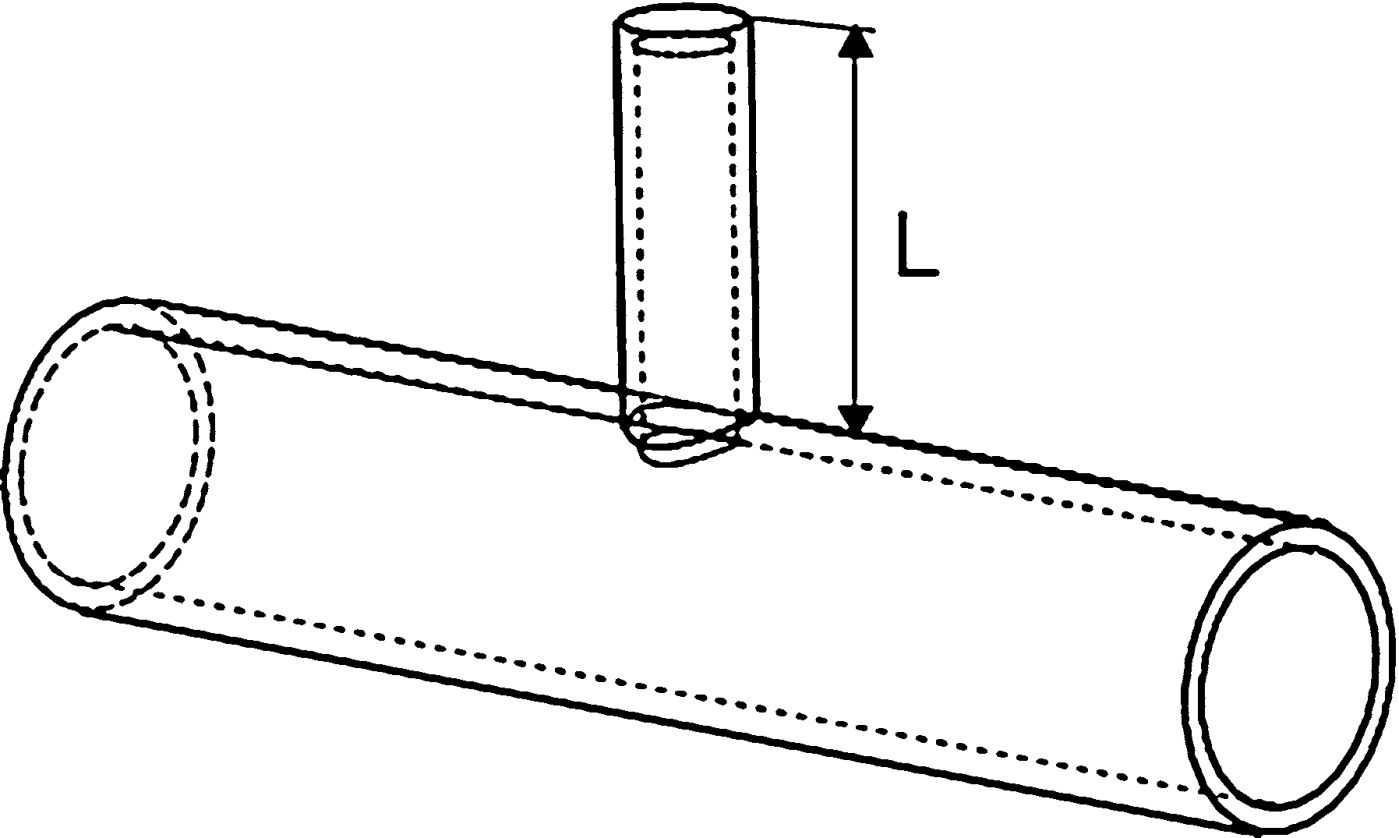
Quarter-wavelength tube

Quarter-wavelength tube is a one-side-closed branch tube installed onto the main pipeline, as shown in Fig. 1. Part of the acoustic wave comes into the bypass tube from the main pipeline, and then reflects back through the closed-end. This offsets the sound wave with the same frequency and opposite phase; this is how the silencer works. In the air inlet system, the quarter-wavelength tube can be used as a single component to eliminate a range of middle-high frequency.

The quarter-wavelength tube’s transmission loss (*TL*) is as follows (Pang et al. 2006):

1 $$TL = 10\log_{10} \left[ {1 + \frac{1}{4}\left( {R\tan \frac{2\pi L}{\lambda }} \right)^{2} } \right]$$

where $L$ is the length of the quarter-wavelength tube (m), $R$ is the ratio of wavelength tube section area and the main tube section area.

When $\frac{2\pi L}{\lambda } = \frac{2n - 1}{2}\pi \left( {{\text{n}} = 1,{ 2},{ 3}, \ldots } \right)$, *TL* tends to be infinity, which means the *TL* reaches the maximum, the bypass tube length is:

2 $$L = \frac{2n - 1}{4}\lambda$$

That means when the wavelength tube’s length is *λ*/4, 3*λ*/4, 5*λ*/4, etc., *TL* reaches the maximum. For the first value being selected (n = 1), the bypass tube’s length is:

3 $$L = \frac{1}{4}\lambda$$

and we have $\lambda = c_{ 0} /f$, where $f$ is the sound frequency (Hz), $c_{0}$ is the speed of sound (m/s). Therefore, the resonance frequency of the quarter-wavelength tube is:

4 $$f = (2n - 1)\frac{{c_{0} }}{4L}$$

From the Eq. 4, we know the resonance frequency of the quarter-wavelength tube merely depends on the length of the tube. The longer the tube, the lower frequency.

One end of the quarter-wavelength tube is open, the other is closed. The sound wave at the opening will flow back and forth like a piston, causing the acoustic radiation impedance. Therefore, the actual working length of the tube will increase. Modified Rayleigh formula (Ruan 2004) is implemented, that is:

5 $$L = L - \frac{8r}{3\pi }$$

where $L_{{}}$ and $L_{a}$ are the quarter-wavelength tube’s actual length and calculation length (m), respectively, and *r* is the quarter-wavelength tube’s radius (m).

Inserting these factors into Eq. 4, we obtain the modified resonant frequency as follows:

6 $$f = \frac{{c_{0} }}{{4L_{a} }} = \frac{{c_{0} }}{{4(L - \frac{8r}{3\pi })}}$$

In spite of the above model as the simplified approximate model of ideal quarter wave tube model, the resonant frequency of the quarter-wavelength tube is closely relevant to the length of the wavelength tube and the radius of the connecting tube from Eq. 6. And the shape, direction or volume of the wavelength tube has less impact on the resonant frequency.

### Helmholtz muffler model

**Fig. 2:**
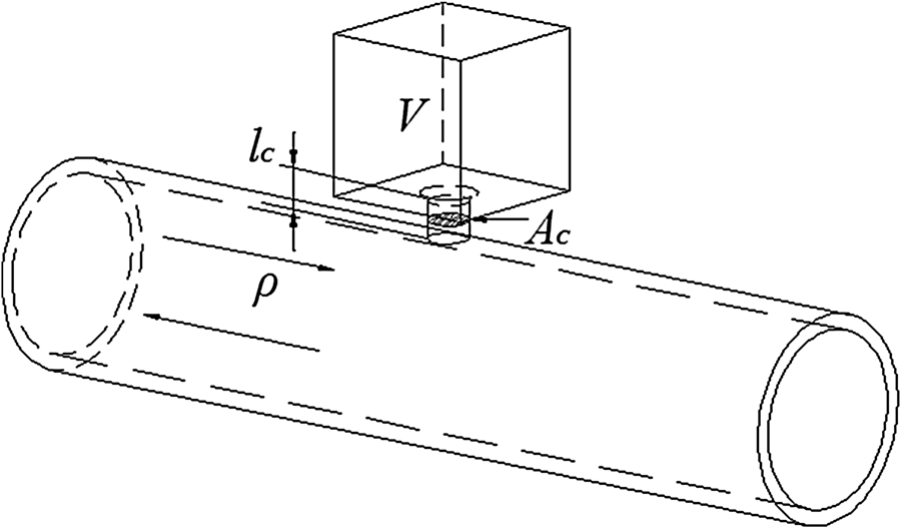
Simplified Helmholtz muffler model

The simplified Helmholtz muffler model is shown in Fig. 2.

The lumped parameter model is expressed as follows:

7 $$m\frac{{d^{2} x}}{{dt^{2} }} + kx = p_{b} A_{c}$$

where *m* is the mass of air in the connected tube, and it can be expressed as $m = A_{c} l_{c} \rho_{0}$, *k* is the elastic coefficient of the resonance cavity, and it can be expressed as $k = \rho_{0} c_{0}^{2} A_{c}^{2} /V$. $A_{c}$ is the section area (m^2^) of the connecting tube, $l_{c}$ is the length of the connecting tube (m), $\rho_{0}$ is the gas density (kg/m^3^), and $V$ is the volume of resonance cavity (m^3^).

Thus, the resonance frequency of Helmholtz muffler is derived using the following equation:

8 $$f_{0} = \frac{{c_{0} }}{2\pi }\sqrt {\frac{{A_{c} }}{{l_{c} V}}} = \frac{{c_{0} r}}{2}\sqrt {\frac{1}{{l_{c} V\pi }}}$$

In spite of the above model as the simplified approximate model of the ideal Helmholtz muffler, the resonance frequency of Helmholtz muffler is closely related to the cross-sectional area and length of connection tube, as well as the volume of the resonance cavity from Eq. 8, while the overall length of the muffler has little influence on the resonance frequency.

Meanwhile, the Helmholtz muffler’s transmission loss is as follows (Pang et al. 2006):

9 $$TL = 10\lg \left[ {1 + \left( {\frac{{\sqrt {A_{c} V/l_{c} } /S_{m} /2}}{{f_{0} /f - f/f_{0} }}} \right)^{2} } \right]$$

where $S_{m}$ is the section area (m^2^) of the main tube.

### New muffler design

**Fig. 3:**
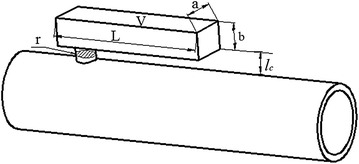
Diagram showing the new muffler. *r* = connecting tube’s radius (m); $l_{c}$ = connecting tube’s length (m); $V$ = volume of resonance cavity (m^3^); $L$ = length of resonance cavity (m); $a$ = resonance cavity width (m); $b$ = resonance cavity’s height (m)

Because the radial size of the quarter-wavelength tube is too large, the tube does not conform with the arrangement of the air inlet system. This paper presents a new muffler structure based on the Helmholtz muffler theory (Fig. 3).

The new muffler consisted of a connecting tube and cavity resonance. It exhibits the functions of both the quarter-wavelength tube and the Helmholtz muffler. The design steps are as follows:

Step 1: Try to determine the value of the connecting tube’s radius $r$. Considering the acoustic radiation effect in the nozzle and convenience to manufacture, the connecting tube’s radius $r$ is recommended for 6-8 mm (The diameter of main tube is 54-96 mm for most of IC engines).

Step 2: According to Eq. 6, the length of the new muffler can be obtained:

10 $$L \approx \frac{{c_{0} }}{{4f_{0} }} + \frac{8r}{3\pi }$$

Step 3: Try to determine the value of the connecting tube’s length $l_{c}$. Since the air in the connecting tube is considered as a lumped mass, the connecting tube’s length $l_{c}$ is not more than half of the wavelength. Taking into account the influence of the tube wall, the connecting tube’s length $l_{c}$ should not be significantly less than its diameter. So the connecting tube’s length $l_{c}$ is recommended for $r \le l_{c} \le 4r$.

Step 4: According to Eq. 8, the volume of the new muffler can be expressed as:

11 $$V = \frac{{c_{0}^{2} r^{2} }}{{4l_{c} f_{0}^{2} \pi }}$$

Step 5: The width and height of the new muffler can be derived as:

12 $$a \times b = \frac{V}{L}$$

Equation 6 is deduced under the assumptions that the geometry of the resonance cavity is significantly less than the acoustic wavelength. It was also assumed that the wave motion and mass distribution conditions in the connection tube and the resonant cavity can be ignored. These hypotheses are hard to meet if the ratios of height and width are too large or too small. In addition, when the ratios of height and width become too small, the transverse wave is dominant in the resonant cavity, and the intersection area of the cavity and connecting tube becomes larger. This leads to strong three-dimensional effects in the intersection area and therefore reduces the accuracy of the calculation.

## Experiment details

**Fig. 4:**
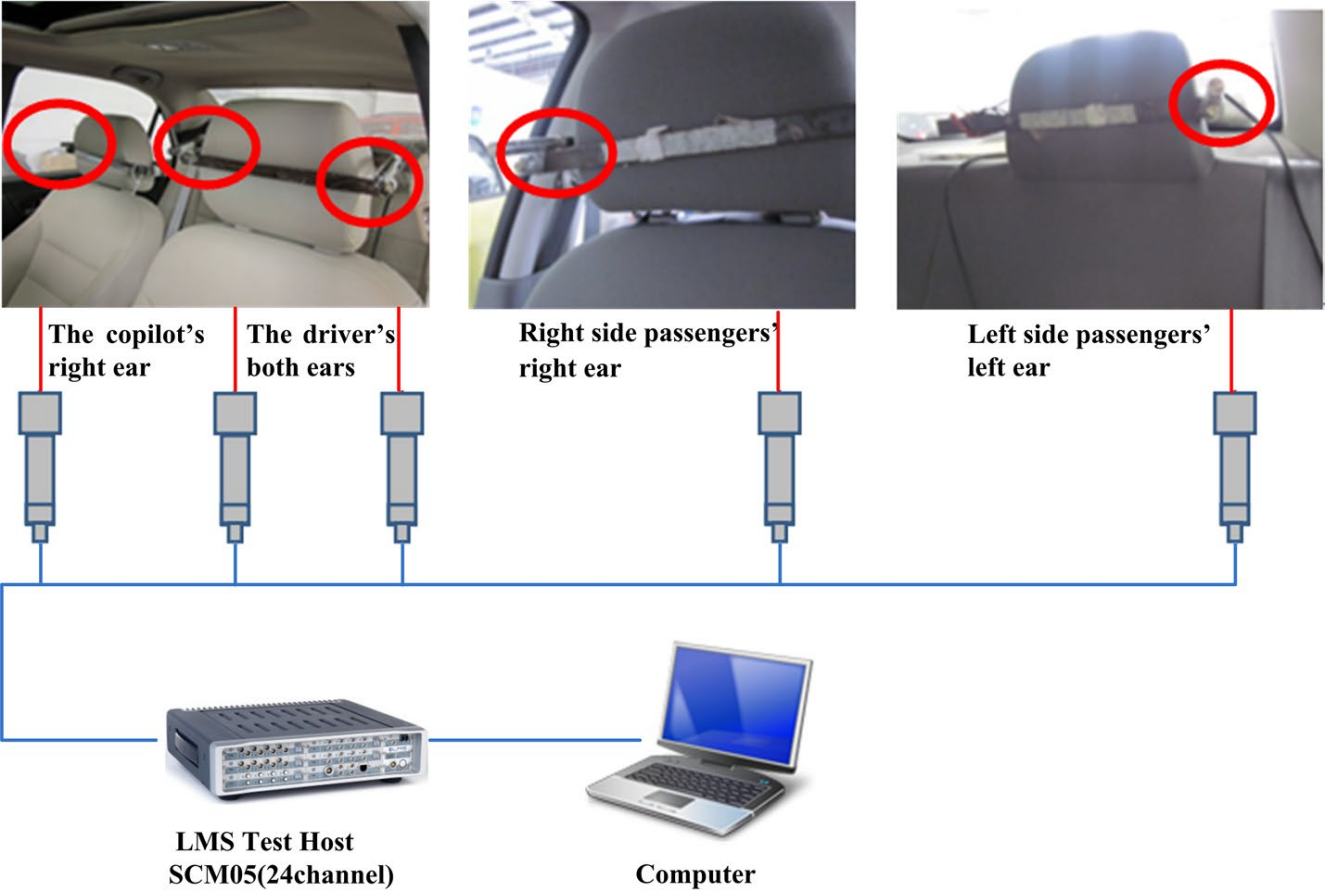
Schematic showing the noise-acquisition system

In order to prove the effectiveness of the new silencer, a vehicle noise test was carried out. The microphone was installed on both of the driver’s “ears”, the copilot’s right ear, and in the backseat as well on the right-side passenger’s right ear, and left-side passenger’s left ear. The microphone’s data wire is connected to the LMS Test Host SCM05, and the data is saved to a computer. Noise data acquisition system’s diagram is shown in Fig. 4.

The noise inside the car consists of the engine intake and exhaust noise, body structure vibrations caused by road, air vibration caused by ground contact by the tires, turbulence noise generated by friction of the car body against the atmosphere, the air-conditioning fan noise, etc. Therefore, interference caused by these extraneous sources must be suppressed to focus specifically on the noise signal which is mainly composed of the engine intake and exhaust noise. Thus, we designed the following experiment: First, the test vehicle was preheated for more than 15 min to complete vehicle preheating until the engine oil temperature reached 90 °C. Second, the air conditioning fan and all the windows were closed. Third, the driver shifted the tested vehicle’s gearbox into second gear, maintaining a speed of only 15 km/h in order to reduce noise generated by air turbulence. Fourth, we chose a smoothly paved driving surface to prevent vehicle structure noise and tire noise, and all the researchers inside the car remained silent. The data acquisition system was then activated. The driver gradually pushed the accelerator pedal to the floor, and kept the throttle valve wide open until the engine speed reached 5000 rpm/min. After maintaining this engine speed for 2 s, the accelerator pedal was released. Finally, the noise data acquisition system was deactivate at completion of the experiment. The test conditions are presented in Table 1.

**Table 1:** Vehicle testing conditions

Testing condition	Testing state
The normal temperature	15 °C
The starting speed	15 km/h
Full-throttle acceleration	Engine speed 1000–5000 rpm full-throttle acceleration in second gear
The test distance	200 m

## Results and discussion

### Experiment analysis

The resulting curve of the vehicle prototype’s in-car noise analysis is shown below.

**Fig. 5:**
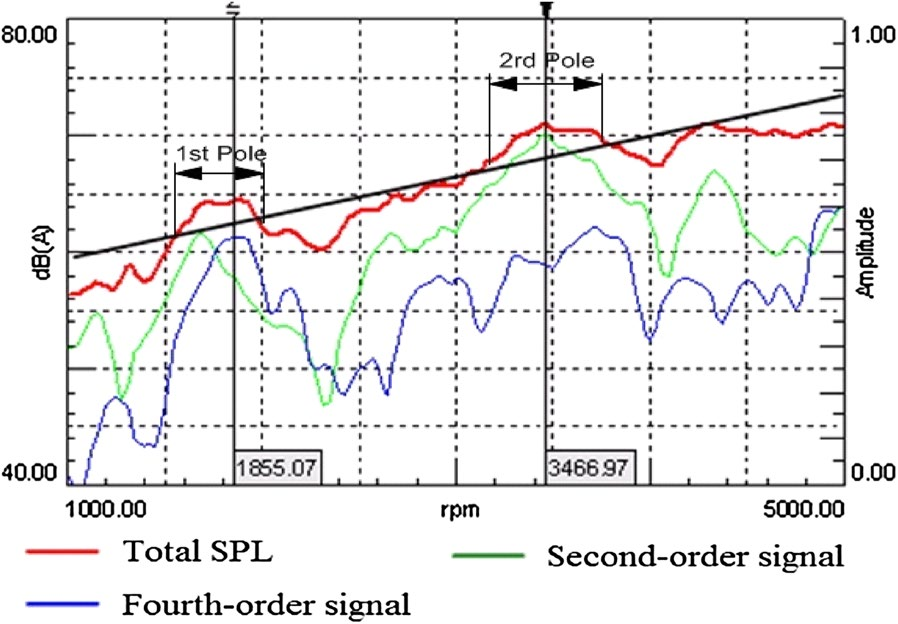
Speeding car noise curve. *SPL* sound pressure level

As shown in Fig. 5, the black slash stands for the tested vehicle’s in-car noise level under the condition of second gear full throttle acceleration. It was observed that when the engine speed was in the range of 1000–5000 rpm, the noise ranges measured from 60 to 75 dB(A), and noise peaks emerged when the engine speed reached 1900 and 3500 rpm. The engine’s fourth order component takes main contribution to the noise peak at 1900 rpm, and the peak ranges from 1600 to 2000 rpm. Simultaneously, the engine’s second order component mainly contributes to that at 3500 rpm, and the peak ranges from 3200 to 3750 rpm. Except for these two noise peaks at 1900 and 3500 rpm, the SPL of intake noise increased linearly with the rising of engine speed. The difference among the engine’s second order components, and the total SPL was more than 5 dB(A), the same as forth order components. It indicated that sound pressure of each frequency in intake noise is uniform, and the engine noise performance was satisfactory. Therefore, the paper aims to design a muffler which can eliminate the noise peak when the engine speed is at the range of 1600–2000 and 3200–3750 rpm.

Because the engine’s dynamic air noise had the same frequency as the engine ignition, we can apply the following equation to calculate the engine air inlet noise frequency:

13 $$f_{e} = \frac{\omega z}{ 6 0i} \times 2k_{o}$$

where $\omega$ is the speed of the engine, *z* is the number of engine cylinders, *i* is the engine stroke coefficient (for four stroke is 2), $k_{o}$ is the harmonic number (order number), and $f_{e}$ stands for the noise frequency.

We then obtain,

$$\begin{aligned} & f_{e1} = \frac{\omega z}{ 6 0i} \times 2k_{o} = \frac{ 1 9 0 0\times 4}{ 6 0\times 2} \times 2 \times 4= 507\;{\text{Hz}} \\ & f_{e2} = \frac{\omega z}{ 6 0i} \times 2k_{o} = \frac{3500 \times 4}{ 6 0\times 2} \times 2 \times 2 = 467\;{\text{Hz}} \\ \end{aligned}$$

**Fig. 6:**
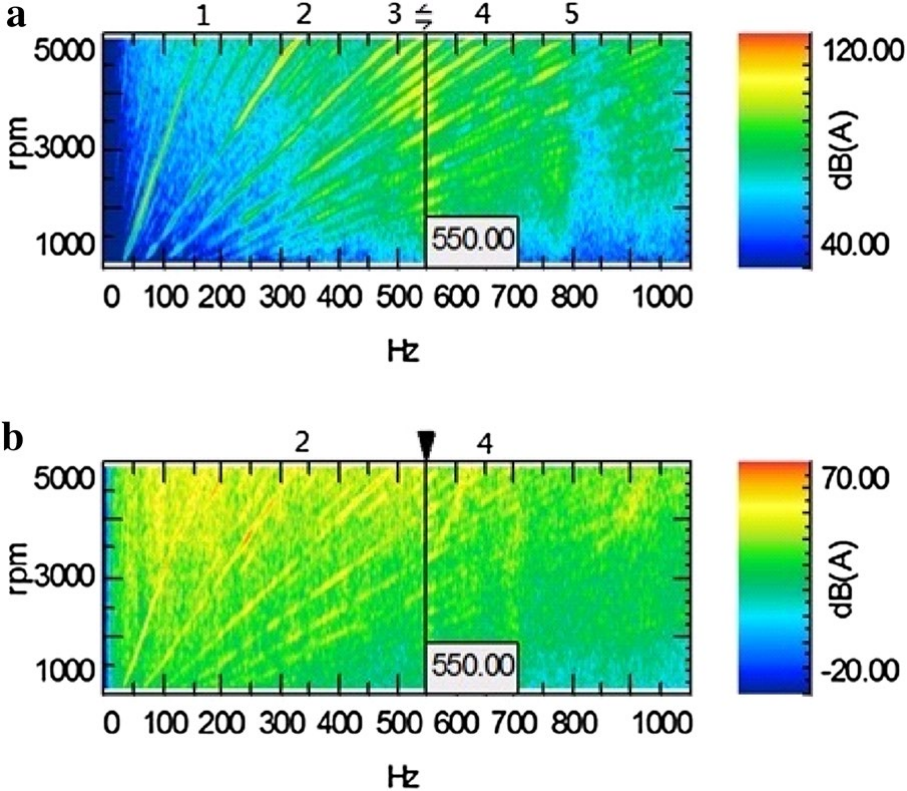
Comparison of air inlet and the in-car noise color map. (The *vertical axis* is the engine speed, the *horizontal axis* is the noise frequency, the SPL is expressed by the color.). **a** Air inlet noise color map, **b** in-car noise color map

By the comparison and analysis of the air inlet noise color map (Fig. 6a) and the speeding in-car noise color map (Fig. 6b), we find these two maps match each other quite well. It indicates that intake noise is one of the main sources of in-car noise, and reduction of the intake noise can decrease the in-car noise. There are ray lines in bright color of Fig. 6a. They illustrate the SPL of the different order noise of engine in the process of accelerating. The ray of 1st, 2nd, 3rd, 4th and 5th order noise are highlight, and the SPL is significantly higher than that of the rest of the region. Moreover, the sound pressure of the 2nd order and 4th order noise are maximum (shown in bright yellow or even orange). Furthermore, noise frequency at the range of 470–570 Hz exist a highlight area, the noise of 3rd, 3.25th, 3.5th, 3.75th, 4th, 4.25th, 4.5th, 5th and 6th orders are the most bright light, which means their sound pressures are maximum. Compared with Fig. 6b, both the resonance peaks are found at about 550 Hz, and the results agree with the theoretical calculation from Fig. 5. There is a clear correlation; therefore, we conclude that peak noise is caused by poor matching of the air inlet system with the acceleration process. There is a resonance peak for air inlet noise at 470–570 Hz, which causes the excitation of the resonance frequency band at 470–570 Hz for the in-car noise.

### Examples and solutions

Because the noise energy is too high at the range of 470–570 Hz, we designed a new muffler with a wide noise suppression frequency band.

Assuming the quarter-wavelength tube resonant frequency is 490 Hz, and considering the speed of sound is 340 m/s, and connecting tube radius $r = 8{\kern 1pt} \;{\text{mm}}$, then we obtain,

$$L = \frac{{c_{0} }}{{4f_{0} }} + \frac{8r}{3\pi } = 180\;{\text{mm}}$$

Assuming the Helmholtz silencer resonant frequency is 570 Hz, and the connecting tube length is 20 mm, we then obtain,

$$V = \frac{{c_{0}^{2} r^{2} }}{{4l_{c} f_{0}^{2} \pi }} = 9 0 , 6 5 0\;{\text{mm}}^{3}$$

Then:

$$a \times b = \frac{V}{L} = 503\;{\text{mm}}^{2}$$

Size: $$a = 25\;{\text{mm}}$$, $$b = 20{\kern 1pt} \;{\text{mm}}$$

**Fig. 7:**
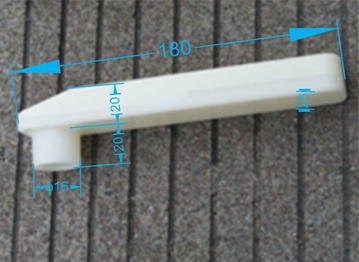
Photograph of the new muffler

The new muffler and its main dimensions are listed in Table 2, and Fig. 7 shows its actual object.

**Table 2:** Main dimensions of new muffler

Muffler part	Size (mm)
The connecting tube ($r$ × $l_{c}$)	8 × 20
The resonance cavity (L × a × b)	180 × 25 × 20

### Validation and discussion

**Fig. 8:**
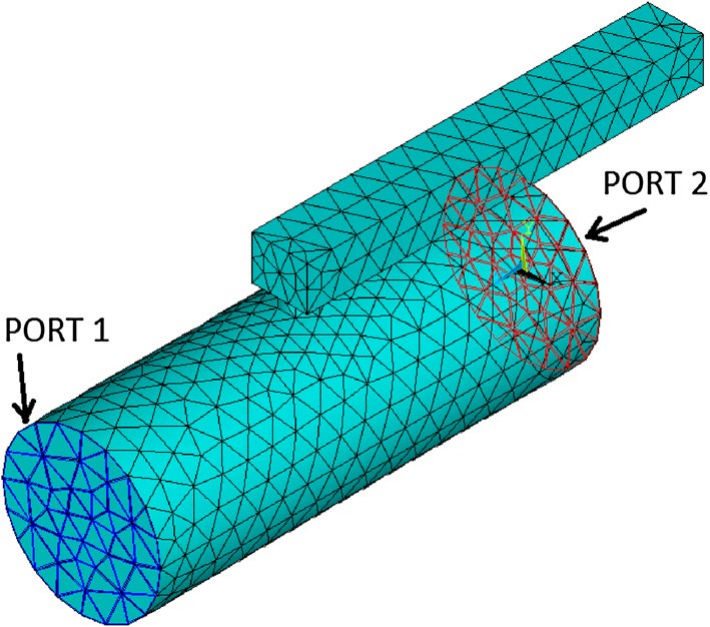
Grid graph

The frequency spectrum of the new muffler is difficult to obtain by the theoretical calculation. So the Finite Element Method (FEM) was used to calculate the transmission loss. The details of the model are as follows:

Air mass density: $\rho_{air} = 1.2041\;{\text{kg/m}}^{ 3}$; air sound speed: $c_{0} = 343.24\;{\text{m/s}}$, main pipe diameter: $d_{m} = 0.076\;{\text{m}}$; element type: FLUID221. PORT 1 is inlet port; PORT 2 is outlet port as shown in Fig. 8.

**Fig. 9:**
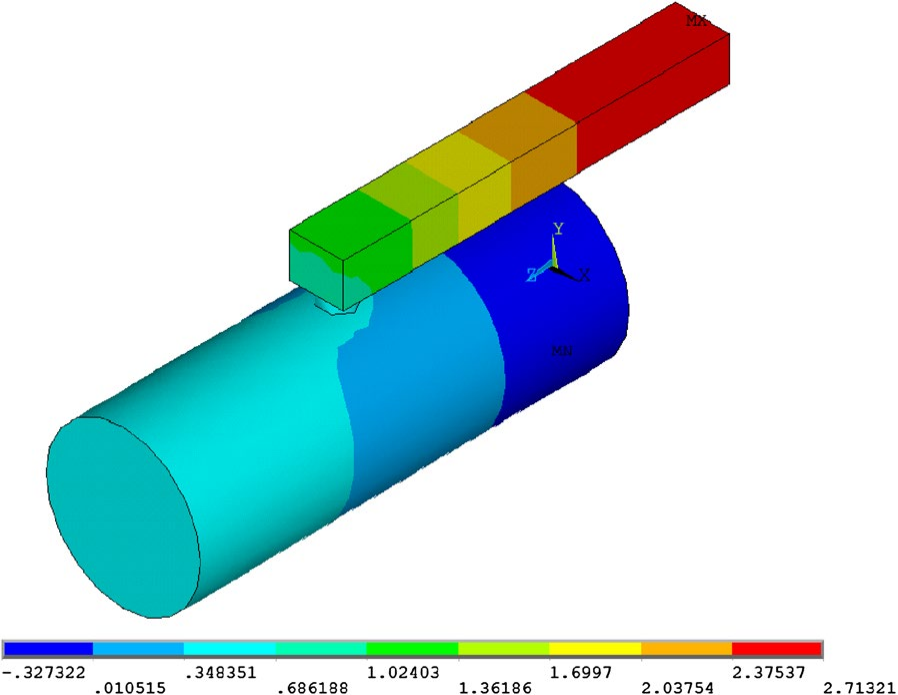
Pressure contours in the muffler (f = 516 Hz)

**Fig. 10:**
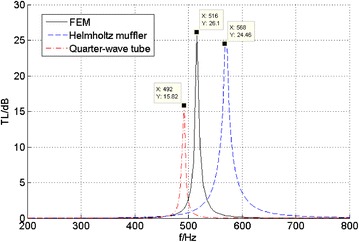
The transmission loss of the new muffler by three methods

Figure 9 shows the pressure contours in the muffler when the noise frequency is 516 Hz. The transmission loss of the new muffler by FEM is presented in Fig. 10 with the results calculated using a quarter-wavelength tube and Helmholtz muffler respectively by using Eqs. 1 and 9. It can be observed that the transmission loss of the new muffler has a wider frequency band of noise elimination than quarter-wavelength tube, and it is similar to Helmholtz muffler. However, the radial size of the new muffler is only 40 mm, which is significantly less than the regular Helmholtz muffler. The new muffler has a good anechoic effect in the 470–550 Hz range.

**Fig. 11:**
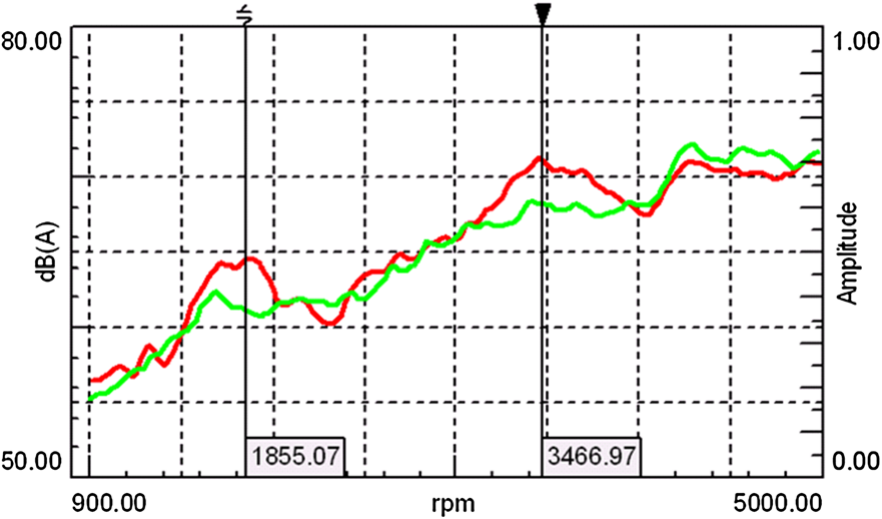
In-car noise curve during acceleration before (*red*) and after (*green*) installing the new muffler

The acceleration data of the in-car noise was recorded during the speed test and was later analyzed, as shown in Fig. 11. Compared with Fig. 5, the peak value of 1855 and 3467 rpm in the graph corresponds to that of the 4th order and 2nd order signal of the engine respectively. By Eq. 13, the two engine speeds corresponds to the noise frequency of 494.7 and 462.3 Hz, which are nearby the anechoic frequency band from 470 to 550 Hz. After installing the new muffler, the peak noise value vanishes at 3500 rpm. Also, the sound pressure level at 1900 rpm was reduced, and the curve of sound pressure level-rotation rate became a bit smoother. The noise with the muffler is slightly louder than that without the muffler at 5000 rpm. The reason is that the air intake of the IC engine increases with the speed of revolution. When the high speed air flows through the bypass cavity of the muffler, the muffler causes the sound pressure to increase under the influence of the unstable airflow and the acoustic geometry of the cavity. It means the noise caused by the airflow excites the acoustic mode of the muffler, which result in the coupling between the airflow and the noise. However, this kind of the muffler noise has little effect on driving due to the IC engine rarely reaching the rotational speed above 4000 rpm during operation. So the characteristics of noise, vibration, and harshness in the air inlet system are improved with the installation of the new muffler.

## Conclusion

In this paper, we focused on the problem of automobile engine low frequency noise and compare several existing muffler design methods, such as the theory of the quarter-wavelength tube and the Helmholtz muffler. We then designed a new type of muffler. After testing its performance in a real-world high speed vehicle, we report the following findings and conclusions:

1. This new silencer consists of a connecting tube and a unidirectional asymmetric resonance cavity. The muffler exhibits the advantages of both the quarter-wavelength tube and the Helmholtz muffler. The new muffler has a wider frequency band of noise elimination than the quarter-wavelength tube, and it is similar to the Helmholtz muffler. However, the radial size of the new muffler is significantly less than the regular Helmholtz muffler.
2. The new muffler’s noise elimination frequency has been derived via a calculation method and FEM method proposed in this study. The size of the muffler’s connection tube was selected based on the diameter of the intake tube, and then according to the frequency calculation, we derived the specific optimal geometric dimensions of the cavity resonance muffler.
3. The experimental data shows that there is a wide noise elimination frequency band around the calculated noise frequency. The new type of muffler design presented here may be used in the air intake system of some types of cars, thus improving the driving experience of both driver and passengers.
